# Multi-target intervention mechanisms and prospects of the traditional Chinese medicine Scutellaria baicalensis georgi in Alzheimer’s disease

**DOI:** 10.3389/fphar.2026.1707688

**Published:** 2026-02-26

**Authors:** Minghao Zhao, Yang Qu, Siqi Zhang, Miao Zhang, Huihui Wang, Yubo Yang, Guocheng Xue, Xuantong Hou, Xinyu Yan

**Affiliations:** 1 Graduate Schools, Heilongjiang University of Traditional Chinese Medicine, Harbin, Heilongjiang, China; 2 Acupuncture and Moxibustion 10 Departments, The Second Affiliated Hospital of Heilongjiang University of Traditional Chinese Medicine, Harbin, Heilongjiang, China

**Keywords:** Alzheimer’s disease, anti-inflammatory, baicalein, baicalin, brain-gut axis, cell death, neuroprotection, traditional Chinese medicine

## Abstract

Alzheimer’s disease (AD) is one of the most prevalent central nervous system disorders affecting middle-aged and elderly populations. As a neurodegenerative disease, its primary clinical manifestations include memory impairment, cognitive dysfunction, and behavioral abnormalities. However, there are limited clinically available treatments for AD. Existing medications neither cure the disease nor halt its progression, and are often associated with significant side effects. *Scutellaria baicalensis Georgi*, with its long history of medicinal use, shows potential for treating central nervous system disorders. Modern pharmacological research has revealed its antioxidant, anti-inflammatory, antiviral, neuroprotective, and immunomodulatory properties. Its active metabolites, such as baicalin and baicalein, exert multi-target effects by simultaneously influencing Aβ production and aggregation, tau phosphorylation, and microglial activation, while also regulating brain-gut axis function. This systematic review examines the mechanisms of action of baicalin and baicalein, the active metabolites of *Scutellaria baicalensis Georgi*, in treating Alzheimer’s disease, offering novel insights and research directions for modern medical approaches to Alzheimer’s disease treatment.

## 1 Introduction

Alzheimer’s disease (AD) has emerged as a major public health challenge in the 21st century. According to 2023 statistics from the World Health Organization, there are over 55 million AD patients worldwide, with projections indicating this number will exceed 152 million by 2050—far surpassing the combined total of cancer and cardiovascular disease cases (GBD, 2019 Dementia Forecasting Collaborators, 2022; Zheng and Luo, 2025).

**FIGURE 1:**
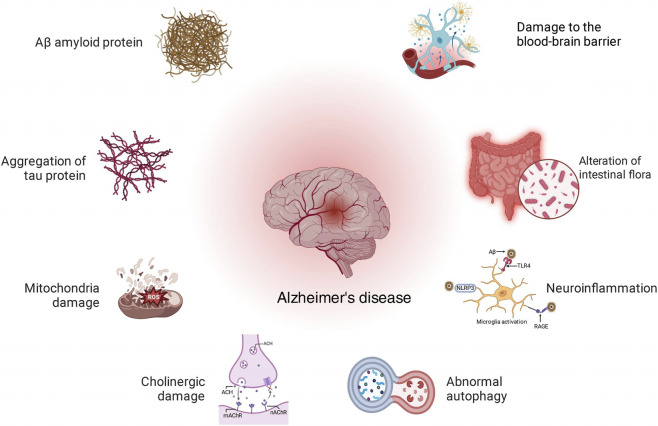
Diagram for the pathogenesis of AD.

Alzheimer’s disease is a progressive neurodegenerative disorder primarily characterized by cognitive decline, particularly memory impairment, along with diminished orientation, judgment, and problem-solving abilities. Additionally, patients may experience language dysfunction, personality changes, and behavioral and psychiatric symptoms (such as depression, anxiety, hallucinations, etc.) (Abdelnour et al., 2022). The etiology of Alzheimer’s disease is complex and multifaceted. Beyond the key roles of beta-amyloid (Aβ) plaques and tau protein (Tau), neuroinflammation (Beata et al., 2023), Autophagy-lysosomal pathway (Kerr et al., 2017), Mitochondrial Function (Shoshan-Barmatz et al., 2018), Cholinergic transmission (Stanciu et al., 2019), Various factors, including oxidative stress and genetic susceptibility, may influence the progression of AD (Bateman et al., 2012; Bai et al., 2022). These intertwined factors collectively contribute to the progression of AD (Figure 1).

Current first-line medications (such as acetylcholinesterase inhibitors and NMDA receptor antagonists) only alleviate some symptoms without significantly altering disease progression and are often accompanied by side effects including dizziness and gastrointestinal reactions (Shi et al., 2016; Durmaz et al., 2023) Against this backdrop, developing novel AD treatments with fewer adverse effects and multi-targeted mechanisms has become a key research focus. Simultaneously, natural metabolites are regarded as promising candidate sources due to their polypharmacological properties.

Scutellaria baicalensis Georgi is a perennial botanical drug belonging to the genus Scutellaria within the Lamiaceae family. It features fleshy rhizomes, branched stems, and lanceolate or linear-lanceolate leaves, with purple-red or blue corollas. Native to several Southeast Asian countries, it thrives in sunny, drought-tolerant conditions and adapts well to sandy soils. The Compendium of Materia Medica explicitly documents Scutellaria’s applications in treating diarrhea, dysentery, hypertension, hemorrhage, insomnia, inflammation, and respiratory infections (Zhao et al., 2016).

Baicalin (BI) and its aglycone baicalein (BE) are the primary flavonoid metabolites found in the root of *Scutellaria baicalensis Georgi* (Liang et al., 2017). BI and BE exhibit anticancer activity, hepatoprotective effects, broad-spectrum antibacterial and antiviral properties, and potent antioxidant capabilities (Zhao et al., 2016; Dinda et al., 2017; Wang R. et al., 2024). Recent studies have indicated that BI and BE exhibit significant neuroprotective effects on neurons and brain tissue (Sabry et al., 2024).

**FIGURE 2:**
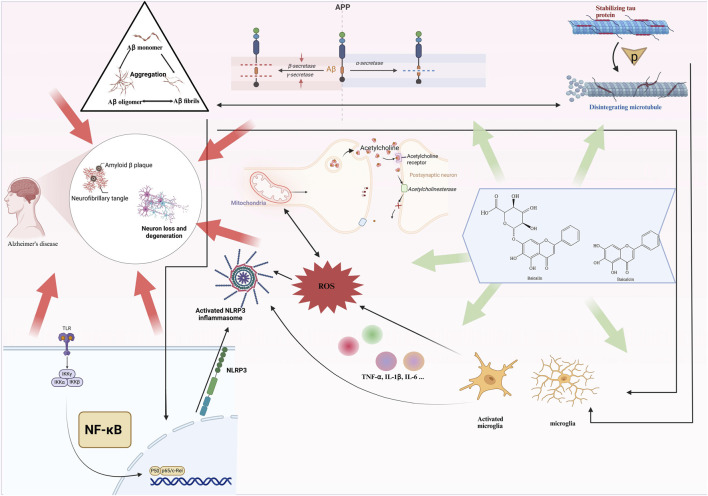
Baicalin and baicalein targeting Alzheimer's disease: key mechanisms of action.

Given the multifaceted roles of BI and BE in treating neurodegenerative diseases, this review summarizes their primary mechanisms of action in AD therapy (Table 1) (Figure 2), aiming to provide reference evidence for future research and explore their potential value in clinical applications.

**TABLE 1:** Mechanisms of baicalin and baicalein in combating Alzheimer’s disease.

Compound	Concentrations	Model	Molecular mechanisms	References
Baicalein	30, 50 μM	SH-SY5Y cells, Hela cells	Inhibit the fibrillation and oligomerization of Aβ1–42	Lu et al. (2011)
Baicalein	10 mg/kg	Tg2576mice	To promote nonamyloidogenic processing of amyloid precursor protein and reduce production of β-amyloid	Zhang et al. (2013)
Baicalin	5, 10, 20, 30, and 40 μM	SH-SY5Y cells	By inhibiting the Ras-ERK signaling pathway and down-regulating the expression of CyclinD1 protein, the cell cycle progression of neurons is suppressed, thereby reducing the generation of Aβ and its neurotoxic effect on nerve cells	Song et al. (2022)
Baicalein	40 and 80 mg/kg	APP/PS1 mice	Inhibiting the activity of GSK3β, reducing the level of Aβ protein, inhibiting Tau phosphorylation, and restoring spine density	Gu et al. (2016)
Baicalin	20 mg/kg	3xTg-AD mice	Interfere with the activities of cyclooxygenase-2 (COX-2) and 5-lipoxygenase (5-LOX), inhibit the activation of the NLRP3 inflammasome, and block the activation of transcription factors such as nuclear factor κB (NF-κB), thereby reducing the deposition of p-Tau and Aβ	Bitto et al. (2017)
Baicalin	1 mg/mL	BV2 cells, SH-SY5Y cells	Suppression of activation of the NLRP3 inflammasome and the TLR4/NF-κB signaling pathway	Jin et al. (2019)
Baicalin	2.5, 7.5 , 22.5 μM	BV-2 cells	Inhibition of the TLR4/MyD88/NF-κB signal transduction pathway mediated by TLR4	Li et al. (2022)
Baicalein	50 and 100 mg/kg/	VD rats	Attenuated hippocampal inflammatory responses by inhibiting the activation of the TLR4/MyD88/NF-κB signaling pathway	Song et al. (2024)
Baicalin	103 mg/kg	C57BL/6 mice	Activation of SIRT1, inhibition of HMGB1, and reduction of microglial activation, leading to decreased levels of inflammatory cytokines	Li et al. (2020)
Baicalin	50, 100 μM	BV2 cells	Regulation of the JAK2/STAT3 signaling pathway to inhibit Aβ-induced microglial activation and its inflammatory response	Xiong et al. (2014)
Baicalin	0.5, 5 μM and 30, 60 mg/kg	HT-22 cells Male C57 mice	Regulation of the PDE-PKA-Drp1 signaling pathway to alleviate memory deficits and mitochondrial damage induced by amyloid β oligomers	Yu et al. (2022)
Baicalein	10, 20, 40 mg/kg	ICR mice	Inhibition of PDE2 and PDE4 activity elevates intracellular levels of cAMP and cGMP, promotes BDNF expression, and ameliorates Aβ-induced memory deficits and neuronal atrophy	Shi et al. (2021)
Baicalin	50, 100, 200 mg/kg	Wistar rats	Prevented Aβ-induced decrease in mitochondrial membrane potential, increase in the Bax/Bcl-2 ratio, release of cytochrome c, and activation of caspase-9 and caspase-3	Ding et al. (2015)
Baicalein	30, 100 mg/kg	Wistar rats	Inhibit the generation of reactive oxygen species (ROS) in mitochondria and enhance the efficiency of oxidative phosphorylation, stabilize the mitochondrial membrane potential and alleviate swelling; regulate the apoptotic pathway (upregulate the Bcl-2/Bax ratio, inhibit the release of cytochrome c, and maintain the mitochondrial homeostasis and functional integrity of the brain	He et al. (2009)
Baicalin	10 mg/kg	SD male rats	Reduction in the expression of neuronal pentraxin-1 (NPTX1) and neuronal pentraxin-2 (NPTX2), decreased levels of C-reactive protein (CRP), inhibition of Aβ-induced neuroinflammation, and attenuation of microglial activation and inflammatory response	Zhao et al. (2023)
Baicalein	5, 10 mg/kg	Wistar rats	Inhibition of acetylcholinesterase (AChE) activity and elevation of acetylcholine (ACh) levels enhance cholinergic signaling	Zhou et al. (2016)
Baicalein	10 mg/2 mL	Wistar rats	Inhibition of acetylcholinesterase (AChE) activity and enhancement of nicotinic acetylcholine receptor (nAChRs) expression	Guo et al. (2011)
Baicalein	5, 10, 25, 50 μM	Human neuroblastoma cell line SK-N-MC	Inhibiting the JNK/ERK signaling pathways, restoring intracellular GSH levels, and reducing ROS accumulation	Moslehi et al. (2012)
Baicalein	100, 200 mg/kg	3×Tg-AD mice	Inhibition of CX3CR1/NF-κB signaling was associated with a phenotypic switch of microglia from M1 to M2	Xie et al. (2023)
Baicalein	10, 20, 40 μM 140, 280, 560 mg/kg	C57BL/6 mice BV2 cells	Inhibiting the NLRP3/caspase-1/gasdermin D (GSDMD) pathway to alleviate neuroinflammation	Rui et al. (2020)
Baicalin	60 mg/kg 1, 4, 8 μg/mL	bEnd.3 cells BALB/C mice	This effect promotes the nuclear accumulation of Nrf2 and restores the Nrf2–HO-1/NQO1 antioxidant axis, thereby reducing ROS production and lipid peroxidation burden and enhancing cellular antioxidant capacity	Wang et al. (2021)
Baicalin	0, 50, 100, 200 mg/kg 0, 12.5, 25, 50 μM	BV2 cells C57BL/6 mice	By modulating the Keap1–Nrf2 ubiquitination–degradation axis to enhance Nrf2 activity, downstream antioxidant molecules such as HO-1 are upregulated, thereby reducing ROS accumulation and lipid peroxidation and suppressing NF-κB–mediated activation of the NLRP3 inflammasome	Huang et al. (2024)

This review aims to comprehensively and systematically summarize the current understanding of the molecular mechanisms of baicalin and baicalein in the treatment of Alzheimer’s disease (AD), thereby providing a reliable theoretical framework and evidence-based strategy for their development as AD therapeutics. The literature search methodology is as follows. Our literature screening process was conducted in the PubMed and Web of Science databases. Key search terms included “Alzheimer’s disease,” “baicalin,” “baicalin,” and “therapeutic mechanisms.” Expanded categories included “traditional Chinese medicine,” “neuroprotection,” “antioxidant,” “neuroinflammation,” “β-amyloid,” “tau protein,” “neurodegenerative disease,” “memory impairment,” “synaptic plasticity,” “cholinergic system,” and “brain-gut axis.” Logical operators applied included “AND” (intersection), ‘OR’ (union), and “NOT” (exclusion). Publication dates were restricted to the past 5 years, though we appropriately relaxed this timeframe for studies offering classical perspectives or significant historical value. Additionally, a dedicated screening mechanism was implemented to exclude non-compliant literature, including case reports, experience summaries, monographs, and studies with low-quality evidence.

## 2 Structure and pharmacokinetics of baicalin and baicalein

BI and BE are flavonoid metabolites (Sabry et al., 2024). BI is the glucuronic acid ester of BE, with the chemical formula C_21_H_18_O_11_ (Li-Weber, 2009). The multiple phenolic hydroxyl groups present in the molecule form the crucial structural basis for its antioxidant function. BE, with the chemical formula C_15_H_10_O_5_, serves as the aglycone of BI. Its benzene ring and epoxy structure confer potent anti-inflammatory and antioxidant activity. These two structures are structurally similar and can interconvert with the participation of gut microbiota and enzymatic reactions within the body (Wang J. et al., 2024) (Supplementary Figure S1).

BI and BE are primarily obtained from Scutellaria baicalensis Georgi through methods such as reflux extraction, water extraction, ultrasonic-assisted extraction, supercritical fluid extraction, and enzyme-assisted extraction (Lee et al., 2014; Meng et al., 2025).

BI exhibits high water solubility but relatively weak intestinal epithelial transport capacity, typically requiring specific transporters or enzymatic processes to enter the bloodstream. In contrast, BE molecules are smaller and more lipophilic, enabling easier penetration through intestinal epithelial cell membranes and direct entry into the bloodstream without relying on specific transport mechanisms or enzymatic activation. Consequently, BE demonstrates superior advantages in crossing the intestinal barrier (Zhang et al., 2007; Li et al., 2011).

Pharmacokinetic studies indicate that BE undergoes rapid phase II metabolism (such as glucuronidation and sulfation) following absorption *in vivo*, with corresponding metabolites (e.g., BE-6-O-sulfate) detectable (Zhang et al., 2004). In non-human primate models, the absolute bioavailability of BE administered orally (50–500 mg/kg) was approximately 13.1%–23.0%, exhibiting a decreasing trend with increasing dose (Tian et al., 2012). Xing et al. found in rat studies that the absolute oral bioavailability of BI was only 2.2% (Xing et al., 2005). Experiments by Lai, M.Y. et al. demonstrated that absorption of the parent compound after oral administration of BE is negligible, while its glucuronidated and sulfated metabolites dominate in plasma. Compared to dose-corrected intravenous administration, its absolute bioavailability is 40%. Following oral administration of BI, only glucuronide and sulfate metabolites of BE were detected in plasma, with BI exhibiting a relative absorption rate of approximately 65% compared to BE. The time to peak concentration for BI was significantly delayed relative to BE, and the peak concentration of BE-bound metabolites was markedly reduced, indicating slower absorption and lower extent of absorption for BI (Lai et al., 2003).

Following intravenous injection, BE distributed throughout the lungs, liver, heart, brain, and kidneys. BI primarily distributed in lung tissue, with significantly increased distribution in brain tissue after cerebral ischemia/reperfusion (I/R) injury. Approximately 30 min after intravenous injection of 24 mg/kg BI, the maximum concentration of BI in cerebrospinal fluid (CSF) reached 344 μg/L. The distribution half-life and elimination half-life of BI in the CSF of normal rats were 0.8868 min and 26.0968 min, respectively. In contrast, the distribution half-life and elimination half-life in the brains of I/R model rats were 2.084 min and 34.4998 min, respectively (Huang et al., 2008).

Although multiple experimental results indicate that BE and BI can cross the BBB to some extent (Chen et al., 2016). However, due to its extremely low oral bioavailability, the actual effective drug concentration reaching brain tissue remains very limited.

Additionally, another study indicates that rats with middle cerebral artery occlusion absorb BI more effectively than sham-operated rats (Zeng et al., 2010). These findings suggest that under pathological conditions, BI and BE exhibit superior therapeutic efficacy in brain tissue. Both BI and BE are excreted via the biliary and renal pathways (Akao et al., 2009). In summary, these results collectively indicate that BE and BI undergo significant enterohepatic first-pass metabolism and transport limitations prior to entering the systemic circulation after oral administration, thereby reducing their effective exposure in plasma.

## 3 Therapeutic effects and mechanisms of baicalin and baicalein in Alzheimer's disease

### 3.1 Inhibition of Aβ deposition and tau aggregation

Aβ deposition is a key pathological component in the pathogenesis of Alzheimer’s disease (Bowirrat et al., 2025). Aβ is a short peptide fragment composed of 36–43 amino acids, generated through sequential cleavage of the amyloid precursor protein (APP) by β-secretase (BACE) and γ-secretase. BACE first cleaves APP at a specific site, followed by further hydrolysis by γ-secretase to produce Aβ peptides of varying lengths (O'Brien and Wong, 2011). There are two subtypes of BACE: BACE1 and BACE2. BACE1 is considered a significant drug target due to its critical involvement in Aβ formation (Cai et al., 2001; Cole and Vassar, 2007). Lu, J.H. et al. demonstrated through *in vitro* experiments that BE directly interferes with the self-assembly process of Aβ peptides in a dose-dependent manner, inhibiting the formation of early soluble oligomers while also blocking the generation of β-sheet fibrils (Lu et al., 2011). Han, J et al. demonstrated through computer-simulated molecular docking and *in vitro* cell experiments that BE is a specific inhibitor of BACE1 and exhibits high affinity for the enzyme (Han et al., 2019). This discovery not only reveals a novel molecular mechanism by which BE modulates Aβ metabolism, but also provides a potential direction for developing naturally sourced BACE1 inhibitors. Zhang, S.Q. et al. utilized the Tg2576 mouse model to demonstrate that BE activates gamma-aminobutyric acid (GABA) A-type receptors, promoting the non-amyloid degradation pathway of APP. This increases α-secretase activity, enhances APP cleavage at the α-site, and generates sAPPa with neuroprotective effects. Consequently, Aβ production is reduced, exerting neuroprotective benefits (Zhang et al., 2013).

The excessive phosphorylation of tau protein and its subsequent aggregation into neurofibrillary tangles is another hallmark feature of Alzheimer’s disease (Friedhoff et al., 1998).

Research by Gu, X.H., et al. indicates that long-term oral administration of BE reduces glycogen synthase kinase-3β (GSK-3β) activity by activating the Akt signaling pathway, which induces its inhibitory phosphorylation. As GSK-3β serves as a key upstream kinase mediating abnormal phosphorylation of tau protein, this regulatory mechanism limits the initiation of pathological tau phosphorylation cascades at the mechanistic level. Further site-specific analysis revealed that BE treatment significantly suppressed abnormal phosphorylation of Tau protein at the Ser202/Thr205 site, providing clear molecular evidence for its intervention in Tau-related neuropathological alterations (Gu et al., 2016).

Additionally, research by Bitto, A. et al. indicates that Flavocoxid, a compound containing BI, can regulate pathological modifications of Tau and APP at the molecular level by inhibiting the abnormal activation of stress-related kinase ERK1/2, thereby blocking inflammation- and oxidative stress-driven pro-phosphorylation signaling cascades. Specifically, Flavocoxid treatment significantly suppressed abnormal phosphorylation of Tau at Thr181, thereby mitigating its potential neurotoxicity. Concurrently, Flavocoxid downregulated phosphorylation of APP at Thr668. Dephosphorylation at this site is believed to facilitate APP’s transition from the β-amyloidogenic pathway to the non-amyloidogenic metabolic pathway, mechanistically reducing the propensity for Aβ generation (Bitto et al., 2017).

Although studies by Bitto, A. et al. indicate that Flavocoxid exerts multifaceted beneficial effects in AD models, the drug specificity and mechanism of action remain unclear. Flavocoxid is essentially a compound formulation composed of two natural flavonoids (BI and catechin). Further experimental validation is required to determine which component or target primarily contributes to Flavocoxid’s efficacy.

Overall, The deposition of Aβ is closely associated with tau protein pathological changes, and the two processes reinforce each other (Zhang et al., 2021). Against this backdrop, the aforementioned studies indicate that BI and BE can target key pathological processes such as Aβ production and abnormal phosphorylation of Tau.

### 3.2 Suppression of inflammatory responses

Neuroinflammation plays a multifaceted role in AD, involving mechanisms such as microglial activation, proinflammatory cytokine release, and blood-brain barrier disruption. By promoting neuronal injury, synaptic loss, oxidative stress, and inflammasome activation, neuroinflammation further exacerbates the pathological progression of AD. Consequently, modulating the neuroinflammatory response has emerged as a critical therapeutic target for AD (Scheltens et al., 2021; Ebrahimi et al., 2025).

#### 3.2.1 Role of NF-κB-centric signaling network regulation in glial cell-mediated neuroinflammation

The nuclear factor kappa-B (NF-κB) signaling pathway is a crucial intracellular signaling cascade that regulates various physiological and pathological processes, particularly those involving immune responses, inflammation, cell survival, and apoptosis (Guo Q. et al., 2024). Additionally, experiments by Li, B. et al. revealed that BI inhibits the direct independent activation of myeloid differentiation protein 88 (MyD88) in microglia, thereby reducing miR-155 expression in LPS-stimulated microglia. This further blocks neuroinflammation mediated by the TLR4-MyD88-NF-κB and mitogen-activated protein kinase (MAPK) pathways, downregulating the mRNA and protein expression levels of inducible nitric oxide synthase (iNOS) and cyclooxygenase-2 (COX-2). This ultimately blocks the release of nitric oxide (NO) and prostaglandin E2 (PGE2), thereby reducing cytokine and pro-inflammatory factor levels (Li et al., 2022). In a rat model of vascular dementia induced by chronic cerebral hypoperfusion, BE was demonstrated to reduce glial cell activation and proinflammatory factor release by modulating the TLR4/MyD88/NF-κB signaling pathway, thereby alleviating cognitive impairment caused by neuronal damage in the hippocampal CA1 region (Song et al., 2024).

CX3CR1 is a chemokine receptor specifically overexpressed on the surface of microglia in the central nervous system, with its primary ligand being the membrane-bound or soluble CX3CL1 (Fractalkine) secreted by neurons. This receptor-ligand axis plays a critical role in maintaining bidirectional communication and functional homeostasis between microglia and neurons. By mediating neuron-derived signals that finely regulate microglia, CX3CR1 signaling suppresses excessive microglial activation, modulates their migration, phagocytic activity, and inflammatory cytokine release, thereby limiting inflammatory damage to neurons and synaptic structures (Camacho-Hernández and Peña-Ortega, 2023). Experiments by Xie, X.M., et al. revealed that BE induces a phenotypic shift of microglia from M1 to M2 type by regulating the CX3CR1/NF-κB signaling axis (Xie et al., 2023).

High Mobility Group Box 1 (HMGB1) is a non-histone DNA-binding nuclear protein widely distributed within cells. Within the central nervous system, HMGB1 can induce neurotropism, disruption of the blood-brain barrier, neuroimmune responses, and neuronal death (Bell et al., 2006; Festoff et al., 2016; Fujita et al., 2016). Acetylation of HMGB1 is regulated by the deacetylase sirtuin 1 (SIRT1), which not only promotes autophagy but also exerts protective effects against Alzheimer’s disease (Lee et al., 2008; Cho et al., 2015; Wang et al., 2019). Experiments by Li, Y. et al. demonstrated that BI-dependent activation of siRNA targeting the SIRT1 enzyme suppressed HMGB1 protein secretion in microglia, reduced HMGB1 protein acetylation, and inhibited HMGB1 translocation from the nucleus to the cytoplasm. This decreased HMGB1 binding to TLR4, thereby inhibiting activation of the PI3K/MAPK pathway and downstream signaling, ultimately suppressing the release of TLR4/NF-κB-mediated inflammatory cytokines (Li et al., 2020).

Janus kinase 2 (JAK2)/signal transduction and activation factor 3 (STAT3) signaling is also crucial for cell-mediated inflammatory responses (Fortelny et al., 2024). Proinflammatory cytokines activate JAK2 kinase, prompting rapid phosphorylation of STAT3. Phosphorylated STAT3 then functions as a transcription factor, translocating to the cell nucleus to regulate the expression of multiple proinflammatory cytokines (Zhu et al., 2007). Research by Xiong, J. et al. indicates that BI inhibits JAK2 phosphorylation, thereby reducing STAT3 phosphorylation. Monitoring the microglial activation marker CD11b revealed that BI suppresses microglial proliferation, migration, and the secretion of pro-inflammatory factors (such as IL-6, TNF-α, and NO), ultimately alleviating neuroinflammation (Xiong et al., 2014).

#### 3.2.2 Regulation of the NLRP3 inflammasome pathway by baicalin and baicalein

Activation of the NF-κB signaling cascade serves as the initial triggering event, upregulating NLRP3 and pro-leukocyte interleukin-1β (IL-1β) expression, thereby promoting subsequent NLRP3 inflammasome activation. The NLRP3 inflammasome comprises NLRP3, apoptosis-associated speck-like protein containing a caspase recruitment domain (ASC), and caspase-1. Which activates caspase-1 to cleave and process the inactive precursors pro-IL-1β and pro-IL-18 into mature IL-1β and IL-18 (Juliana et al., 2012).

Research by Jin, X. et al. revealed that BI significantly downregulates the abnormal overexpression of TLR4, inhibits the phosphorylation and degradation of IκBα, blocks the nuclear translocation of the NF-κB p65 subunit, and reduces its transcriptional activity. Further investigations demonstrated that BI significantly reduced NLRP3 protein expression in microglia and brain tissue, inhibited the cleavage and activation of Caspase-1, and reversed the expression of proinflammatory mediators such as IL-1β, IL-18, and iNOS in APP/PS1 transgenic mouse models and LPS/Aβ-induced BV2 microglia (Jin et al., 2019). Experiments by Rui, W., et al. revealed that BE alleviates 1-methyl-4-phenyl-1,2,3,6-tetrahydropyridine (MPTP)-induced neuroinflammation by inhibiting the NLRP3/caspase-1/gasdermin D (GSDMD) pathway (Rui et al., 2020). Additionally, BI exerts antioxidant and anti-neuroinflammatory effects by activating the Nrf2/HO-1 signaling pathway (Wang et al., 2021).

Nrf2-mediated antioxidant effects are crucial for BI’s inhibition of NLRP3 inflammasome activation (Huang et al., 2024).

### 3.3 Antioxidant stress

Due to mitochondrial dysfunction and inflammatory responses, increased production of free radicals such as ROS and an imbalance in antioxidants like superoxide dismutase (SOD) prevent the clearance of excess free radicals. This leads to excessive oxidative stress in neurons, triggering neuronal apoptosis and causing brain nerve damage, which further exacerbates the progression of Alzheimer’s disease (Martínez Leo and Segura Campos, 2019).

Nuclear factor erythroid 2-related factor 2 (Nrf2) is a key transcription factor regulating the induction of antioxidant genes (Vomhof-Dekrey and Picklo, 2012). Experiments by Ding, H. et al. demonstrated that BI restores the activity of SOD, catalase (CAT), and glutathione peroxidase (GPx) by promoting the nuclear translocation of Nrf2 and activating the downstream antioxidant response element (ARE) pathway. This process also upregulates their gene expression, further inhibiting Aβ-induced mitochondrial membrane potential decline and reducing oxidative stress. catalase (CAT), and glutathione peroxidase (GPx) activity while upregulating their gene expression. This further inhibits Aβ-induced mitochondrial membrane potential decline, reduces the Bax/Bcl-2 ratio, blocks activation of caspase-9 and caspase-3 (members of the caspase family), and decreases cytochrome c release (Ding et al., 2015). Concurrently, BE demonstrated significant mitochondrial protective effects in a rat model simulating chronic cerebral hypoperfusion. It reduced mitochondrial reactive oxygen species (ROS) production, elevated membrane potential, promoted oxidative phosphorylation, mitigated mitochondrial swelling, decreased the Bax/Bcl-2 ratio, and inhibited cytochrome c release. These findings indicate its crucial role in maintaining mitochondrial homeostasis and function (He et al., 2009).

Additionally, a study on H_2_O_2_-induced cell death demonstrated that BE effectively blocked intracellular pro-apoptotic processes by inhibiting the activation of the JNK/ERK signaling pathway and maintaining the steady-state levels of the key antioxidant glutathione (GSH) (Moslehi et al., 2012). Substantial evidence indicates that mitochondrial dysfunction represents one of the earliest and most consistent pathological hallmarks of AD, preceding the appearance of overt Aβ and tau pathology (Chou et al., 2011; Jadiya et al., 2019; Teo et al., 2019). Therefore, interventions targeting mitochondrial function are considered a potential therapeutic strategy for AD. Against this backdrop, natural products that regulate mitochondrial dysfunction are increasingly attracting widespread attention.

### 3.4 Enhancing neuronal synaptic plasticity and inhibiting neuronal apoptosis

Synaptic dysfunction is an early event in AD and is closely associated with cognitive decline. BI and BE protect synaptic function and enhance neuroplasticity. Research by Gu, X. H. et al. indicates that BE prevents Aβ42 oligomer-induced damage to long-term potentiation (LTP) by activating Akt phosphorylation and inhibiting the activity of key enzymes such as 12/15-lipoxygenase and GSK-3β. Furthermore, BE restores normal dendritic spine density and reverses memory deficits in Alzheimer’s disease model mice (Gu et al., 2016).

Yu, H. Y. et al. experimentally demonstrated that BI inhibits phosphodiesterase 4 (PDE4), increases cyclic adenosine monophosphate (cAMP) protein levels, further activates protein kinase A (PKA) protein, promotes phosphorylation of dynamin-related protein 1 (Drp1) at Ser637, and inhibits Drp1-mediated mitochondrial fission. significantly elevates microtubule-binding protein levels in neurons, enhances synaptic plasticity, and improves memory deficits (Yu et al., 2022). Additionally, experiments by Shi, J. et al. demonstrated that BE inhibits PDE2 and PDE4, modulates the cAMP/cGMP-pCREB-BDNF pathway, thereby reversing Aβ-induced neuronal atrophy in the hippocampal CA1 region and mitigating damage to synaptophysin and PSD95 proteins (Shi et al., 2021).

Regarding the regulation of synaptic structural proteins and synapse-associated receptors, the neuronal pentamer receptor (NPR) is a membrane-associated protein primarily localized at excitatory synapses. It exhibits particularly high expression levels in the cerebral cortex, hippocampus, and cerebellum (Dodds et al., 1997).

Overexpression of neuron-specific pentaxin 1 (NPTX1) is believed to exacerbate synaptic damage and activate neuronal apoptosis pathways (such as elevated expression of pro-apoptotic proteins Bax and caspase-3); conversely, reduced levels of neuron-specific pentaxin 2 (NPTX2) are closely associated with medial temporal lobe atrophy and memory impairment (Gómez de San José et al., 2022; Vazquez et al., 2025). They can bind to their receptors at excitatory synapses in the central nervous system, promoting the recruitment and aggregation of AMPA-type glutamate receptors, thereby participating in the regulation of synaptic plasticity. These NPTX proteins are considered key mediators of Aβ-induced synaptic loss, ganglion cell damage, and neuronal death (Kirkpatrick et al., 2000; Selkoe, 2002).

Zhao, J. K., et al. observed the expression of NPTX1 and NPTX2 in brain tissue structures through immunohistochemistry and immunofluorescence staining in their study of BI intervention in AD mice. Compared to the AD model group, rats in the AD + BI group showed improved memory and spatial learning abilities, a significant increase in hippocampal neuron count, markedly reduced neuronal apoptosis, along with a significant decrease in NPTX1 expression and a significant increase in NPTX2 expression. This indicates that BI can delay the progression of AD by enhancing synaptic plasticity and reducing neuronal apoptosis (Zhao et al., 2023). This study employed only observational methods such as immunohistochemistry and immunofluorescence staining, without further investigation into molecular mechanisms or gene expression regulation. Consequently, our understanding of the underlying mechanisms remains limited.

The toxicity of Aβ induces abnormal activation of the cell cycle in terminally differentiated neurons (Chao et al., 2019). Abnormal activation of the Ras-ERK pathway can trigger apoptosis, a process closely associated with Alzheimer’s disease (Lee et al., 2009). Cyclin D1 is a protein encoded by the human *CCND1 gene*. It plays a crucial role in regulating the activity of cyclin-dependent kinases (CDKs). Cyclin D1 activates CDKs, enabling them to phosphorylate target proteins and drive progression through the cell cycle (Ding et al., 2020). Cyclin D1 is regulated by the Ras-ERK pathway, and its accumulation and abnormal cell cycle activation are considered to be directly associated with neuronal apoptosis (Malik et al., 2008). Experiments by Song, Z., et al. demonstrated that BI reverses the Aβ1-42-induced entry of SH-SY5Y cells into the S phase by suppressing the activation of the Ras-ERK signaling pathway and reducing Cyclin D1 levels. This treatment increases the number of SH-SY5Y cells in the G2/M phase, decreases the level of abnormally activated neuronal cell death, and mitigates the damage caused by Aβ aggregation to neurons (Song et al., 2022).

### 3.5 Regulating cholinergic levels

The cholinergic hypothesis posits that cognitive decline in Alzheimer’s disease is closely linked to significant degenerative loss of cholinergic neurons in the basal forebrain, reduced acetylcholine synthesis, and impaired cholinergic neurotransmission (Hampel et al., 2018). It has been confirmed that the severity of dementia in Alzheimer’s disease correlates positively with the degree of cholinergic impairment (Ferreira-Vieira et al., 2016; Hampel et al., 2019). Cholinesterase inhibitors can alleviate the symptoms of Alzheimer’s disease, and anticholinesterase medications are currently among the most widely prescribed drugs for treating AD (e.g., Donepezil, Galantamine, Rivastigmine) (Mufson et al., 2008).

Additionally, Aβ exhibits extremely high affinity for the α7 subunit of the nicotinic acetylcholine receptor (nAchR), and this binding has been identified as a critical step for Aβ entry into neurons. This interaction not only significantly reduces nAchR expression but also triggers increased Aβ accumulation and toxicity within neurons. According to Li-li Guo et al., BE improves synaptic nAchR function by upregulating the expression of nAchR α4 and α7 subunits. Following BE treatment, acetylcholinesterase (AchE) and butyrylcholinesterase (Buche) levels decreased by approximately 25% and 18%, respectively, suggesting BE may mitigate Aβ-related pathological changes by modulating the cholinergic axis (Guo et al., 2011).

In this experiment, no significant changes were observed in cholinergic-related mRNA levels, suggesting that regulation primarily occurs at the level of protein translation or stability. In an AlCl_3_-induced AD mouse model study by Zhou, L et al., BE significantly increased acetylcholine (ACh) levels and downregulated AChE activity (Zhou et al., 2016). Although this experiment did not delve into specific mechanisms of action, merely detecting ACh content and AChE activity via colorimetric methods, it is noteworthy that under conditions of co-administration with carbamylcholine (0.3 mg/kg), BE (10 mg/kg) demonstrated superior efficacy in elevating ACh levels and inhibiting AChE activity. This finding suggests BE holds potential advantages in modulating the cholinergic system, indicating significant research value and merit for further investigation.

## 4 Baicalin and baicalein improve AD by regulating brain-gut axis function

**FIGURE 3:**
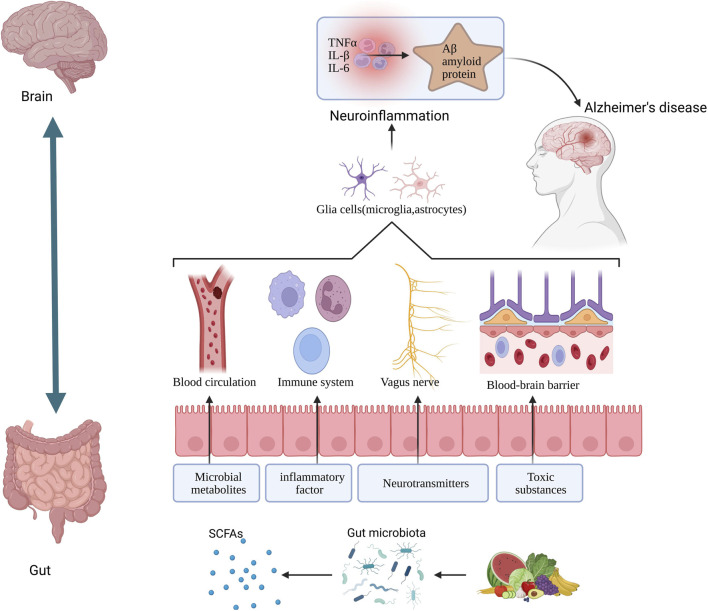
The impact of gut microbiota-derived short-chain fatty acids on Alzheimer's disease pathology.

The gut-brain axis represents a bidirectional communication network connecting the central nervous system and the gastrointestinal system. Information exchange between the gut and the brain primarily occurs through two pathways (Figure 3) (Sohrabi et al., 2022; Kuźniar et al., 2024). One mechanism involves humoral regulation through signaling molecules in the blood, such as metabolites produced by gut bacteria and inflammatory cytokines. The other operates within the autonomic nervous system by mediating interactions between neurons and other cells via neural pathways, including neurotransmitters. Recent studies have indicated a strong association between AD and the gut microbiota. For instance, Koga-Batko et al. reported a significant correlation between dysbiosis in the gut microbiota of AD patients and the onset of the disease (Koga-Batko et al., 2025).

Metabolic products of gut microbiota are various small molecules produced by the fermentation of dietary metabolites and host secretions by gut bacteria, including short-chain fatty acids (SCFAs), tryptophan-derived metabolites, and others (Ji et al., 2025).

These metabolites influence AD through multiple mechanisms. For example, SCFAs help maintain the integrity of the blood-brain barrier, regulate brain energy metabolism, and can interfere with Aβ formation (Braniste et al., 2014; Chen et al., 2022). Butyrate exerts anti-inflammatory effects by inhibiting NF-κB signaling, thereby reducing inflammatory responses in the brain (Sun et al., 2020). Acetate inhibits excessive activation of microglia and the production of the pro-inflammatory factor IL-1β, reduces COX-2 levels, suppresses Aβ-induced activation of the JNK/ERK/NF-κB signaling pathways, and simultaneously upregulates the expression of G protein-coupled receptor 41 (Liu et al., 2020a; Erny et al., 2021). Additionally, gut symbionts such as *Bacteroides* and Bifidobacterium can metabolically produce neuroactive substances like GABA and dopamine. These microbially derived neuroactive metabolites play a crucial role in the gut-brain axis by regulating the host’s mood, behavior, and cognitive functions (Barrett et al., 2012; Dinan and Cryan, 2017).

It is evident that alterations in the gut microbiota play a significant role in the onset and progression of Alzheimer’s disease (AD). In the future, regulating the gut microbiota composition of AD patients through pharmacological interventions or other means, combined with other therapeutic approaches, holds promise as a novel intervention strategy for AD.

BI and BE, as plant-derived antimicrobial metabolites, exhibit significant gut-protective effects, with their physiological mechanisms potentially linked to the regulation of the gut microbiota (Lin et al., 2022). Liu, J. et al. reported that repeated cerebral ischemia-reperfusion significantly disrupts gut microbiota homeostasis in mice, leading to reduced Firmicutes and increased Bacteroidetes. Bifidobacterium intervention effectively reverses these changes and restores microbial balance, with the high-dose (100 mg/kg) group showing superior efficacy compared to the low-dose group (Liu et al., 2020b). In experiments demonstrating the damage caused by ionizing radiation to the intestinal system and microbiota of mice, radiation induced massive apoptosis of intestinal epithelial cells and intestinal mucosal injury, leading to a significant reduction in the abundance of beneficial bacterial groups such as *Bacteroides* and *Lactobacillus*. Compared to the control and irradiated groups, mice treated with intraperitoneal injections of BE (100 mg/kg) exhibited restored abundance of *Bacteroides* and *Lactobacillus* species in their intestines to near-normal levels. Furthermore, BE significantly reduced radiation-induced intestinal cell apoptosis by inhibiting the p53 signaling pathway (Wang et al., 2020). Additionally, BE demonstrated a corrective effect on dysbiosis in a mouse model of trinitrobenzenesulfonic acid-induced ulcerative colitis. BE-treated mice exhibited a significantly increased ratio of Firmicutes to Bacteroidetes, along with markedly elevated levels of beneficial short-chain fatty acids (SCFAs) such as butyrate, propionate, and acetate (Zhu et al., 2020).

*In vitro* microbial culture experiments simulating different pH conditions of the gastrointestinal tract demonstrated that BI and BE significantly promoted the growth of Bifidobacteria under acidic, weakly acidic, and alkaline environments (corresponding to gastric pH = 1.5–2.0, small intestinal pH = 5.5–6.0, and colonic pH = 7.5–8.0, respectively) (Dmitrieva et al., 2023). BI and BE act as prebiotics that adapt to the intestinal environment, thereby helping to improve the composition of the gut microbiota.

Although the aforementioned studies successfully induced gut microbiota dysbiosis and tissue damage using pathological and ionizing radiation models, respectively, these models fail to fully reflect the unique gut microenvironment and microbial composition characteristics specific to AD patients. Therefore, their relevance to the disease requires further validation. Moreover, the molecular regulatory mechanisms by which BI and BE act on the gut microbiota remain unclear. Changes in microbial composition alone cannot fully explain their biological effects. Future studies should integrate metabolomics and host signaling pathway research to elucidate these mechanisms in depth. Nevertheless, BI and BE can improve central nervous system function, reduce Aβ-induced neuroinflammation, and maintain BBB integrity by modulating the gut microbiota and its metabolites. This provides novel insights and potential directions for gut microbiota-mediated AD treatment.

## 5 Discussion

Alzheimer’s disease has been demonstrated to be a multifactorial neurodegenerative disorder, yet most currently approved clinical treatments still target only a single pathway (Yoo et al., 2025). Current drug design for AD primarily targets its symptomatic characteristics (Table 2). Traditional research and therapeutic strategies have largely centered on cholinergic dysfunction, which has long constituted the core approach in AD drug development (Sharma et al., 2019). Cholinesterase inhibitors (such as Donepezil, Galantamine, Rivastigmine) and the NMDA receptor antagonist memantine are often associated with gastrointestinal or neurological adverse reactions (Li et al., 2019). These drugs primarily modulate neurotransmitter systems, with efficacy limited to the early stages of the disease. Anti-Aβ monoclonal antibodies approved in recent years (such as Lecanemab and Donanemab) have expanded the therapeutic intervention pathways for Alzheimer’s disease to some extent. By clearing amyloid plaques from the brain, they can slow cognitive decline by approximately 25%–35% in patients with early-stage AD (Sims et al., 2023). However, these remain single-target strategies primarily focused on extracellular Aβ pathology, with limited impact on key AD pathways such as tau pathology propagation, neuroinflammation, and mitochondrial damage. Additionally, antibody therapies face significant safety limitations, particularly amyloid-related imaging abnormalities (ARIA), including cerebral edema and microbleeds (e.g., ARIA-E incidence was approximately 12% for Lecanemab versus about 2% in the placebo group) (Honig et al., 2024). Therefore, developing therapeutic strategies with broad target coverage, complex mechanisms of action, and improved safety profiles remains a critical need.

**TABLE 2:** Approved pharmacotherapy for Alzheimer’s disease: Mechanisms, efficacy, and safety profiles.

Drug	Mechanism of action/Target	Adverse effects	Clinical positioning	References
Donepezil	Donepezil selectively and reversibly inhibits AChE, prolonging the action time of ACh between synapses, thereby synergistically enhancing the signals mediated by mAChR and nAChR	Mostly gastrointestinal events; overall good tolerability	Symptomatic treatment (cholinergic pathway)	Cacabelos (2007), Modrego et al. (2010), Howard et al. (2012)
Galantamine	Galantamine has dual effects of inhibiting AChE and modulating the conformation of nAChR. It prolongs the half-life of ACh in the synaptic cleft and enhances the central cholinergic tension, thereby strengthening the signals mediated by mAChR and nAChR	Gastrointestinal events, generally manageable	Symptomatic treatment	Hager et al. (2014), Metz and Pavlov (2021)
Rivastigmine	Rivastigmine is a slow and reversible dual cholinesterase inhibitor that can simultaneously inhibit the activities of both AChE and BuChE. Unlike selective AChE inhibitors such as Donepezil, Rivastigmine has a higher affinity for the G1 subtype of AChE, which is mainly distributed in the cholinergic synaptic regions of the hippocampus and cortex. This makes it more likely to enhance the central cholinergic signal transmission rather than the peripheral nervous system, thereby theoretically improving cognitive function and neuroprotective benefits	Transdermal patch reduced systemic AEs; minor local skin reactions	Symptomatic treatment; transdermal formulation improves adherence	Cacabelos (2007), Dhillon (2011), Aguiar et al. (2014)
Memantine	Memantine is an antagonist of the NMDA type glutamate receptor. It improves normal neuronal processing by reducing the activity of NMDA receptors outside the synapse, and it also has affinity for the α7 nAChR receptor	Well-tolerated; adverse events similar to placebo	Symptomatic and disease progression–slowing therapy	Reisberg et al. (2003), Schneider et al. (2011), Bali et al. (2025)
Donanemab	Anti-Aβ monoclonal antibody targeting N-terminally truncated insoluble amyloid plaques	The risks of ARIA and infusion-related reactions	Disease-modifying therapy (enhances microglial-mediated plaque clearance)	Shcherbinin et al. (2022), Sims et al. (2023)
Lecanemab	Lecanemab exerts its therapeutic effects in early Alzheimer’s disease by selectively binding to and neutralizing soluble amyloid-β protofibrils, thereby reducing their neurotoxicity, inhibiting downstream Tau pathological propagation, and promoting microglial-mediated clearance of aggregated Aβ	The risks of ARIA-E/H and infusion-related reactions	Disease modification (slowing disease progression and clearing amyloid plaques)	Johannesson et al. (2024), van Dyck et al. (2025)

Unlike traditional single-target drugs, BE and BI can synergistically exert effects across multiple key pathological pathways in AD. Existing evidence indicates that BE and BI demonstrate a degree of mechanism convergence with currently approved therapeutic agents in several key pathological pathways associated with Alzheimer’s disease. At the cholinergic system level, both exhibit AChE inhibitory activity, aligning with the core mechanism of donepezil, galantamine, and rivastigmine—inhibiting acetylcholine degradation and enhancing cholinergic neurotransmission. Notably, BI’s dual inhibitory effect on both AChE and BuChE shares high mechanistic similarity with rivastigmine’s mode of action. Regarding nicotinic cholinergic signaling regulation, although BE is not a direct allosteric modulator of nAChRs, it exerts an indirect yet functionally consistent regulatory effect with galantamine in enhancing nicotinic cholinergic signaling by upregulating nAChR expression or improving receptor-associated functional states. Within the glutamatergic system, both BE and BI have been reported to mitigate NMDA receptor-mediated excitotoxicity. This functionally overlaps with memantine’s neuroprotective effects achieved by inhibiting pathological NMDA receptor hyperactivation, despite differences in molecular sites of action and direct targets. Regarding amyloid pathology, BE and BI inhibit Aβ production, interfere with aggregation processes, and reduce amyloid plaque burden. For the key pathological indicator of cerebral amyloid deposition, their therapeutic objectives align to some extent with Donanemab’s immunologically mediated clearance of amyloid plaques, despite differences in specific pathways and intervention levels. Furthermore, existing research indicates that BE and BI activate neurotrophic signaling pathways such as cAMP/CREB–BDNF, which are closely associated with neuronal survival and synaptic plasticity. This pathway is also considered one of the important downstream mechanisms through which donepezil and memantine exert their neuroprotective effects during long-term treatment (Dwamena et al., 2025). This phenomenon suggests that BE and BI may exhibit functional overlap or similar regulatory mechanisms to certain existing clinical drugs in maintaining synaptic function and supporting neuronal survival.

Regarding tau, these two metabolites effectively inhibit excessive tau phosphorylation and promote the disaggregation of fibrillar tangles, thereby reducing tau toxicity—an aspect that most currently approved AD drugs have yet to address. Furthermore, BE and BI broadly suppress neuroinflammation: they modulate the transition of microglia from the M1 inflammatory phenotype to the M2 reparative phenotype, inhibit the release of inflammatory cytokines, and improve the neural microenvironment. Their antioxidant effects, activation of the Nrf2 pathway, and enhancement of mitochondrial energy metabolism enable them to protect neurons at multiple levels, including cellular energy, oxidative stress, and synaptic plasticity. Beyond direct CNS effects, BI and BE may exert neuroprotective actions by modulating the gut-brain axis. They reshape gut microbiota composition, increase beneficial bacteria abundance, and strengthen intestinal barrier function. This promotes short-chain fatty acid production, thereby suppressing increased intestinal permeability and peripheral dissemination of inflammatory signals. Consequently, they enhance blood-brain barrier integrity and ultimately alleviate the inflammatory microenvironment within the CNS. This capacity to regulate the gut-brain axis represents a therapeutic dimension absent in all currently used Alzheimer’s disease medications, further highlighting the unique advantages of BI and BE in multi-targeted interventions.

In terms of safety, BI and BE demonstrate notable advantages. In the relevant experiments of this paper, no significant toxic reactions were observed in animal studies even at higher dosages (e.g., 200 mg/kg). *In vitro* experiments showed no significant cytotoxicity even when the effective concentration was increased to 500 µM. Furthermore, existing studies have not reported any cases of significant toxic reactions or organ damage induced by these metabolites. Overall, BI and BE are generally recognized as having favorable safety profiles, exhibiting low toxicity, and with limited reports of adverse reactions (Li et al., 2021; Oshima et al., 2024). Preliminary human studies also indicate that BE is safe for oral administration and well tolerated (Pang et al., 2016). In contrast, cholinesterase inhibitors commonly cause gastrointestinal adverse reactions, while anti-Aβ monoclonal antibodies carry a higher risk of ARIA. Therefore, BI and BE are more suitable for long-term use or as safe adjuncts in multi-drug regimens for elderly patients.

In exploring combined intervention strategies, the synergistic application of BI and BE with probiotics yields mutually reinforcing effects: on one hand, it enhances the efficacy of probiotics in correcting gut microbiota imbalance; on the other hand, it improves the bioavailability and therapeutic efficacy of BI and BE through probiotic-mediated optimization of the intestinal environment, thereby promoting the absorption of beneficial substances by the gut (Guo F. et al., 2024). Additionally, oral antibiotics have been shown to cause significant disruption to the gut microbiota (Charitos et al., 2025). Experiments by Noh, K. et al. demonstrate that when used in combination with antibiotics, BI and BE can upregulate beneficial gut microbiota such as lactobacilli and bifidobacteria, reduce intestinal damage, and enhance the antibacterial efficacy of antibiotics within the body (Noh et al., 2016). For patients with Alzheimer’s disease, the combined use of BI and BE offers a novel research perspective as a therapeutic strategy when antibiotics are administered to treat systemic inflammation and probiotics are employed. Furthermore, studies have demonstrated that BE exhibits synergistic effects when combined with Memantine (Jadhav and Kulkarni, 2023). This indicates its potential for further research and application within multi-mechanism collaborative intervention strategies.

Although numerous animal studies indicate that these two metabolites can prevent the progression of AD pathology by regulating multiple molecular signals and exerting diverse pharmacological effects, making them promising natural products for treating AD, current research on BI and BE for AD treatment still has several shortcomings. First, existing pharmacological studies are primarily confined to animal experiments, with significant limitations in research paradigms. Some animal studies fail to specify the purity of BE and BI used or lack purity reference data, undermining the credibility of findings. Experimental designs also require greater rigor. Research emphasis is overly concentrated on acute-phase neuroprotective mechanisms, often lacking long-term follow-up observations. Consequently, there remains insufficient evidence supporting the role of these metabolites in the long-term prognosis of AD. In numerous animal studies, BE and BI across different dose gradients (low, medium, high) are compared only longitudinally against model control groups, lacking lateral efficacy comparisons with currently clinically used AD treatments (e.g., Donepezil, Galantamine, Rivastigmine). This unidimensional experimental design prevents establishing the relative efficacy ranking of BE and BI compared to other treatment options through evidence-based medicine. Furthermore, existing studies generally lack systematic documentation of key AD clinical symptoms and quantitative analysis of the duration-dependent effects of BE and BI interventions. Overall, the scientific quality of current primary research on BE and BI for AD treatment remains low, leaving no evidence to suggest their superior efficacy over existing interventions. Notably, despite these limitations, numerous animal studies have demonstrated that BE and BI can indeed improve AD-induced cognitive impairment through multiple mechanisms, positioning them as promising candidate drugs for combating AD. Based on these findings and challenges, we recommend conducting more rigorous, multi-tiered research to clarify the role of BE and BI within the AD treatment framework. Specifically, a four-tier validation system could be established, comprising: 1. Randomized Controlled Trials: Incorporate positive control drugs in animal models or preclinical studies, then directly compare the efficacy of BE and BI against standard therapeutic agents (e.g., approved drugs like donepezil) through randomized controlled trials to evaluate their relative efficacy and safety. 2. Multi-omics mechanism validation: Construct an integrated multi-omics (genomics, proteomics, metabolomics, etc.) analysis platform to deeply decipher the molecular mechanisms and targets through which BE and BI act on AD pathological processes, validating their pharmacological pathways at the systems level. 3. Multi-center clinical trials: Conduct multi-center clinical trials to evaluate the clinical translational value of BE and BI, supported by preliminary safety and efficacy evidence. This will help determine their effective dosage range, efficacy, and potential adverse reactions in AD patients, providing a basis for clinical application. 4. Long-term efficacy tracking: Conduct long-term follow-up observations of models and patients receiving BE and BI treatment to evaluate their sustained impact on the AD disease process, including long-term efficacy and safety monitoring of cognitive function, activities of daily living, and disease progression indicators.

Secondly, a critical analysis of existing evidence indicates that both BI and BE exhibit significant multidimensional pharmacological effects in the prevention and treatment of AD. These two flavonoids derived from Scutellaria baicalensis Georgi do not function through a single mechanism. Current research fails to support the hypothesis that either BI or BE acts against AD via a single independent pathway—such as solely anti-amyloid, anti-tau, antioxidant, or anti-inflammatory effects. Conversely, extensive animal model studies demonstrate their broad and comprehensive effects in ameliorating AD pathology: not only do they improve memory deficits, reduce cerebral amyloid plaque burden, and regulate abnormal tau phosphorylation, but they also enhance mitochondrial function and synaptic plasticity while promoting neuronal survival. For instance, BE simultaneously inhibits BACE1 and AChE—key targets in AD—highlighting its potential as a multifunctional neuroprotective agent. Different research teams, due to varying experimental design focuses, have highlighted distinct mechanisms: some emphasize the amyloid-clearing effects of BI and BE, while others concentrate on their anti-inflammatory or anti-Tau pathways. However, their results consistently demonstrate coordinated improvements across various pathological markers, suggesting cross-synergistic interactions among these mechanisms. This indicates that the neuroprotective effects of BI and BE against AD are achieved through the integrated regulation of multiple mechanisms, pathways, and targets. Notably, future research should integrate multi-omics analyses (e.g., transcriptomics, proteomics, metabolomics) to identify the core regulatory network nodes of BI and BE in AD prevention and treatment. This will reveal pivotal hubs in their mechanisms of action and explore their unique advantages in AD management. Advanced network pharmacology and bioinformatics approaches have begun to elucidate the multi-target mechanisms of these flavonoid metabolites. This multi-faceted research should be further deepened in the future.

To date, neither animal studies nor clinical research has definitively established the minimum effective concentration or dose-response relationship for BI and BE in treating AD. Consequently, their minimum effective dose and optimal dosing schedule remain unclear. Given the current lack of evidence, there is an urgent need for more rigorously designed clinical trials to validate their therapeutic effects in human populations and systematically evaluate long-term safety. Simultaneously, the low solubility of these metabolites and the limited efficiency of brain delivery remain critical bottlenecks for clinical translation. To enhance bioavailability, stability, and brain-targeted delivery, innovative formulation strategies are required. These include traditional decoctions based on the synergistic effects of Chinese herbal formulas, as well as nanocarrier systems such as liposomes and solid lipid nanoparticles. Optimization of drug delivery routes also warrants significant attention: for instance, intranasally administered BI liposomes can bypass the blood-brain barrier limitations via the nasal-brain pathway, enhancing intracerebral delivery efficiency and demonstrating neuroprotective effects in rats with cerebral ischemia-reperfusion injury (Xiang et al., 2020).

Overall, BI and BE can serve as multi-target candidates for AD, with their multimodal effects potentially overcoming the limitations of single-target strategies. However, their clinical translation still requires high-quality clinical evidence and breakthroughs in delivery technology.

## 6 Conclusion

These findings provide robust experimental evidence supporting BI and BE as natural therapeutic candidates for Alzheimer’s disease, demonstrating their promising translational potential in slowing disease progression and improving cognitive impairment. Through these efforts, BI and BE hold promise to evolve into safe, evidence-based plant-derived drugs or functional interventions, offering novel approaches and strategies for multi-target comprehensive treatment of Alzheimer’s disease.
