# Supplementary figures and images for: Multi-target intervention mechanisms and prospects of the traditional Chinese medicine Scutellaria baicalensis georgi in Alzheimer’s disease
Source: Front Pharmacol. 2026 Feb 26;17:1707688. doi: 10.3389/fphar.2026.1707688 (PMC12979506; doi:10.3389/fphar.2026.1707688)

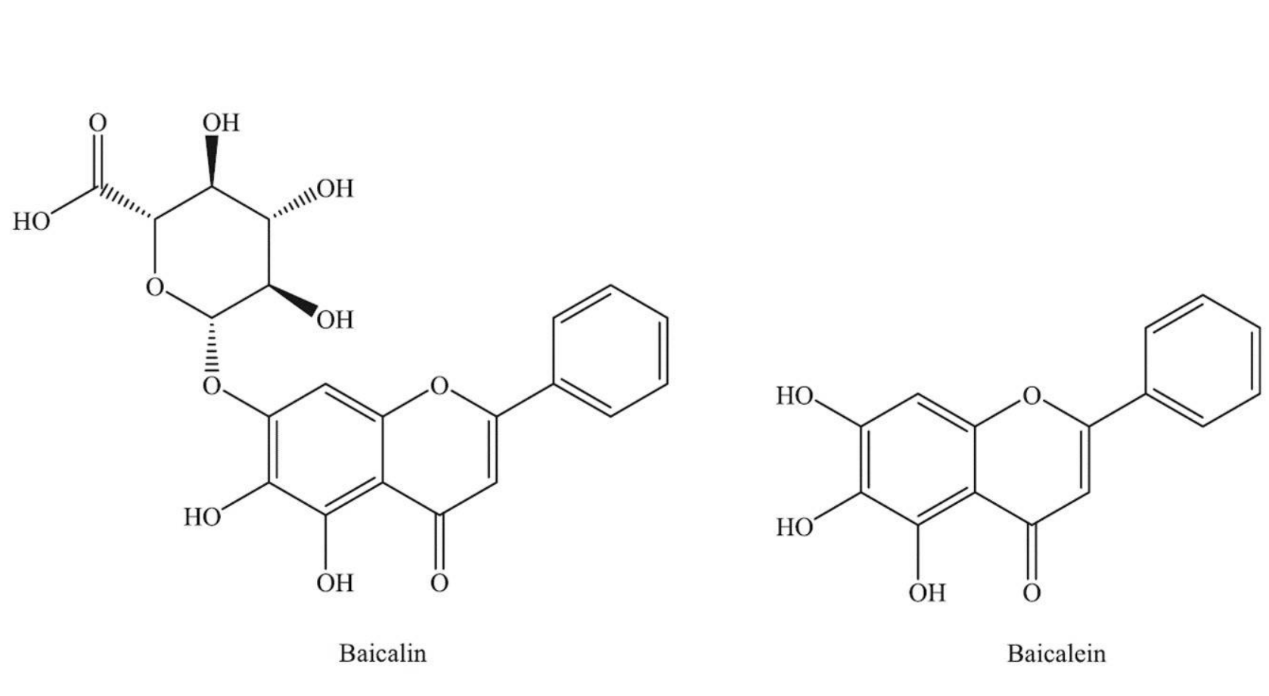

Supplement: Supplementary file 1 [file Image1.png]
